# Nebulized Liposomal Amphotericin B as an Alternative Therapy for an *Aspergillus* Tracheal Plaque

**DOI:** 10.1155/crdi/8467541

**Published:** 2026-04-02

**Authors:** Sofia Valdes-Camacho, Paola Gutierrez-Gallegos, Alanna Barrios-Ruiz, Bryan F. Vaca-Cartagena, Alejandra Yu Lee-Mateus, Isabel Fernandez-Bussy, Kate L. Walsh, Kelly S. Robertson, Sebastian Fernandez-Bussy, David Abia-Trujillo

**Affiliations:** ^1^ Division of Pulmonary, Allergy, and Sleep Medicine, Mayo Clinic Florida, Jacksonville, Florida, USA, mayoclinic.org; ^2^ Pontificia Universidad Católica Argentina, Buenos Aires, Argentina

**Keywords:** bronchoscopy, inhaled liposomal amphotericin B, tracheobronchial aspergillosis

## Abstract

Invasive pulmonary aspergillosis (IPA) typically affects immunocompromised individuals; however, increasing cases in patients without traditional risk factors suggest evolving host susceptibility and predisposing airway pathology. We present a case of an *Aspergillus* tracheal plaque in a 68‐year‐old immunocompetent woman with atypical presentation. Due to adverse effects associated with systemic antifungal therapy, treatment was transitioned to nebulized liposomal amphotericin B (LAmB), resulting in complete plaque resolution without complications. This case highlights the potential role of nebulized LAmB as an alternative treatment for tracheal aspergillosis. Further studies are warranted to refine dosing and expand its clinical applications.

## 1. Introduction

*Aspergillus* is a ubiquitous mold found in decaying organic matter and in the environment, with its ability to cause disease influenced by both pathogen and host factors. While *Aspergillus* infections primarily affect immunocompromised individuals, such as those with malignancies, chronic diseases, posttransplantation status, or on immunosuppressive therapies, [1] cases in patients without traditional risk factors are increasingly identified. Genetic susceptibility may also contribute, as single gene polymorphisms and inborn errors of immunity can increase susceptibility to fungal infection [2]. Clinical manifestations range from localized colonization to invasive disease, with infection occurring in both pulmonary and extrapulmonary sites.

Invasive pulmonary aspergillosis (IPA) can present as tracheobronchitis, characterized by necrotic lesions, ulceration, or plaque‐like pseudomembranes within the tracheobronchial tree [3]. The Infectious Diseases Society of America (IDSA) recommends azoles as first‐line therapy; however, in cases where these are contraindicated or found to be ineffective, alternative antifungal strategies may be required [4]. Liposomal amphotericin B (LAmB), commonly used as prophylaxis in high‐risk patients, has shown promise as a therapeutic option in tracheobronchial aspergillosis [4].

Here, we present a case of pseudomembranous tracheobronchial aspergillosis successfully treated with nebulized LAmB after multiple antifungal therapies failed to achieve clinical improvement.

## 2. Case Description

**FIGURE 2:**
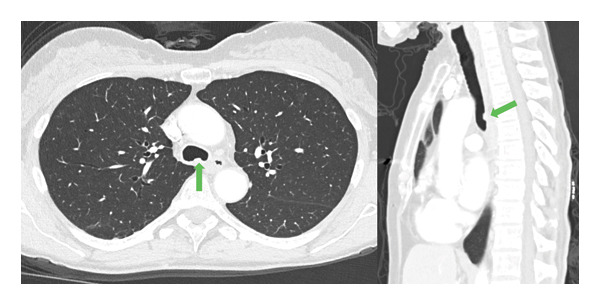
Axial (a) and sagittal (b) chest computed tomography (CT) showing a posterior protrusion (green arrows) in the distal trachea near the carina.

A 68‐year‐old female with a history of expiratory central airway collapse status posttracheobronchoplasty underwent surveillance bronchoscopy due to a nonimproving chronic cough. Bronchoscopic airway examination identified a distal tracheal plaque (Figure 1(a)), which was biopsied with endobronchial forceps and brush, as well as bronchoalveolar lavage (BAL) fluid obtained. Forceps and brushing samples revealed septal fungal hyphae, while *Aspergillu*s antigen (Ag) was ≥ 3.750 on BAL. On a concurrent computed tomography (CT) scan, the plaque presented as a protrusion in the posterior lower tracheal lumen, near the carina, with no evidence of soft tissue infection, extratracheal involvement, or mediastinitis (Figure 2).

FIGURE 1(a) Flexible bronchoscopy showing a pseudomembrane on the posterior tracheal wall, just above the carina. (b) The same lesion was observed 2 months later; the patient was on posaconazole at the time of bronchoscopy.

**Figure:**
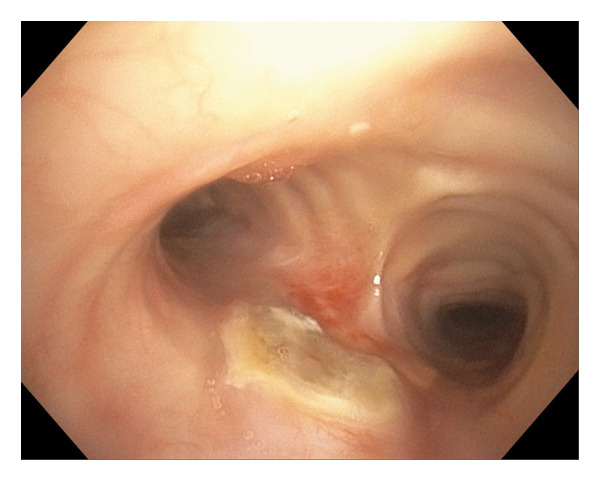
(a)

**Figure:**
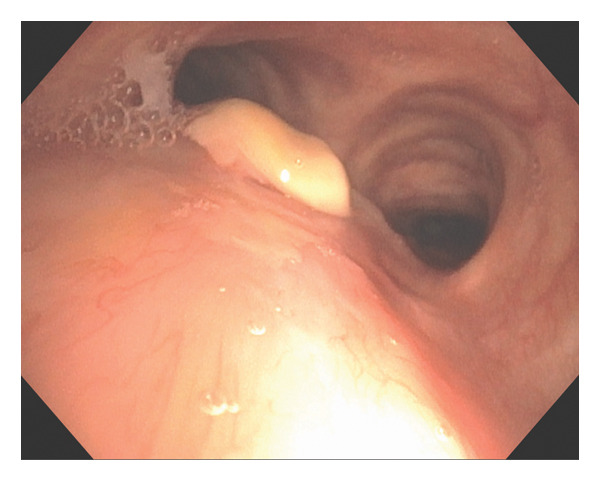
(b)

Over a 3‐month period, multiple antifungals were used. Voriconazole therapy was initiated at 200 mg orally twice daily and increased to 300 mg twice daily; however, despite dose escalation, voriconazole trough blood levels remained subtherapeutic at 0.2 mg/L. Bronchoscopy evaluation during this period showed persistent tracheal plaque (Figure 1(b)). Treatment was switched to posaconazole delayed‐release (DR) 300 mg daily but was ultimately discontinued due to adverse effects, likely related to elevated serum concentrations reaching 4.61 mg/L, prompting consideration of localized antifungal therapy with nebulized LAmB. Optimal dosing was achieved with a 4 mg/mL dilution, and the patient received 12.5 mg (3.5 mL) via nebulization twice weekly. After only three doses administered, over a period of 2 weeks, bronchoscopy confirmed resolution of the fungal plaque, with residual yellow discoloration successfully treated using argon plasma coagulation (Figure 3). Forceps and brushing biopsies reported fragments of benign superficial bronchial mucosa with subepithelial fibrosis with no evidence of malignancy or presence of septal fungal hyphae, while BAL did not show microorganism growth, and *Aspergillus* Ag was below the index level of < 0.5.

FIGURE 3(a) Flexible bronchoscopy demonstrating plaque resolution with residual yellow discoloration. (b) Narrow‐band imaging shows a pink appearance without hypervascularization.

**Figure:**
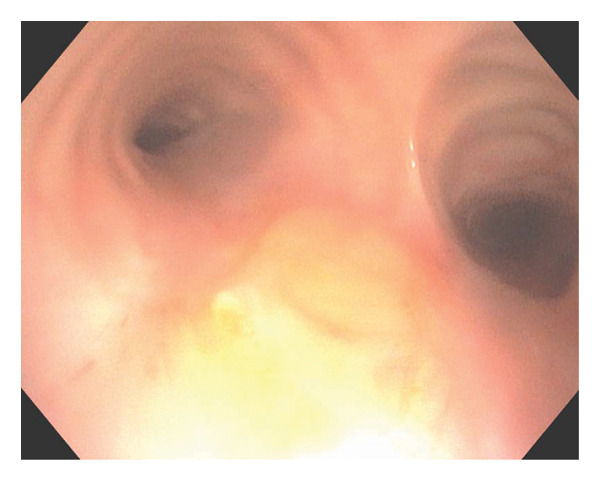
(a)

**Figure:**
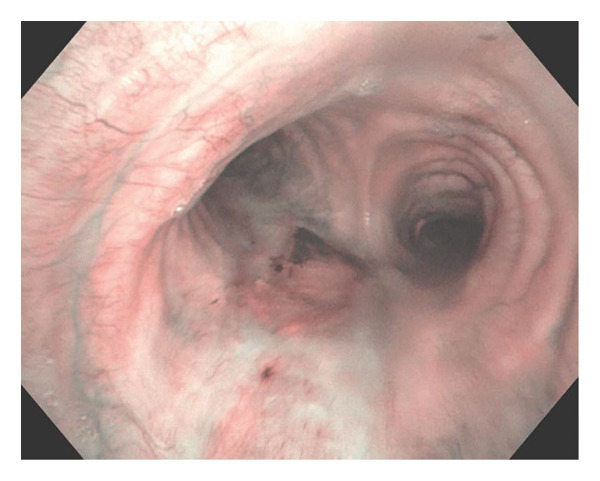
(b)

**FIGURE 4:**
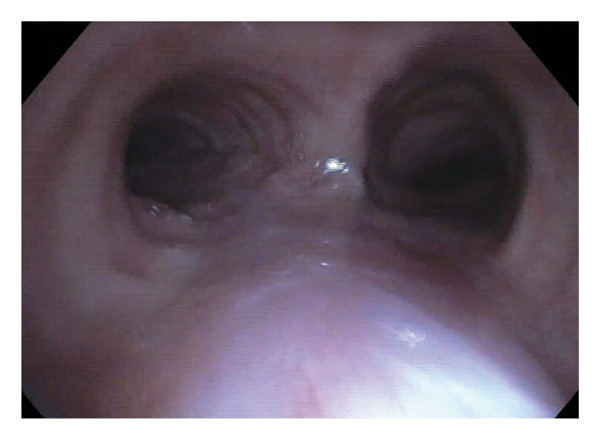
Most recent flexible bronchoscopy view confirming long‐term plaque resolution with mild to moderate bulging of the distal trachea.

The patient continued therapy with nebulized LAmB twice weekly for 4 months. At the most recent follow‐up, 10 months after the initial treatment, routine surveillance bronchoscopy confirmed resolution of the focal protrusion (Figure 4). The patient continues care with Infectious Disease and has been evaluated by Allergy and Immunology for asthma and cough management, with no identified immunodeficiencies or laboratory abnormalities.

## 3. Discussion

While IPA is traditionally associated with immunocompromised states, an increasing number of cases are being reported in immunocompetent individuals or those without traditional risk factors. This shift raises concerns about evolving host susceptibility, environmental changes, or unrecognized predisposing factors [2]. In our patient’s case, laboratory testing, including white blood cell counts and immunoglobulin levels, did not reveal any immunodeficiency. However, her history of underlying lung disease, prior tracheobronchoplasty, and prolonged corticosteroid therapy for asthma may have contributed to airway structural compromise, facilitating *Aspergillus* colonization and invasion. A 2023 Australian study on IPA patients found that while 50% had a hematological malignancy and 7% had a primary comorbidity such as diabetes mellitus or chronic kidney disease, 5% (11 patients) had neither [2]. Notably, 4 of these 11 patients had a history of trauma, surgery, or corticosteroid use, findings that align with our case.

Given its broad‐spectrum antifungal activity, amphotericin B remains a key therapeutic option, particularly in cases where azoles are contraindicated or poorly tolerated, such as in our case report. The patient’s voriconazole blood trough levels were persistently subtherapeutic despite increasing the oral dose to 0.2 mg/L (1–5.5 mg/L) [5]. Conversely, after transitioning to posaconazole, elevated serum concentration levels at 4.61 mg/L (1.5–3.75 mg/L) resulted in adverse effects and discontinuation [6]. The suboptimal effect of systemic therapy led to consideration of localized fungal treatment with nebulized LAmB.

While intravenous amphotericin B deoxycholate has been associated with nephrotoxicity, the development of LAmB and lipid‐based formulations has significantly reduced systemic toxicity [7]. In addition, aerosolized LAmB has been widely used as prophylaxis in lung transplant recipients and patients with prolonged neutropenia, as it allows localized drug delivery to the airway while minimizing systemic absorption and adverse effects [4, 8]. The phospholipid and cholesterol components of lipid formulations share similarities with pulmonary surfactants, enhancing drug deposition in the respiratory tract and making nebulized LAmB an effective option for airway‐targeted antifungal therapy [7].

Precise dosing remains unstandardized, but previously published prophylactic protocols and case reports describe therapeutic use in airway aspergillosis in the range of 2–5 mg/mL; in our patient, optimal dosing was achieved at 4 mg/L [7–10]. The risk of chemical pneumonitis appears low, particularly with liposomal formulations, and reported adverse effects are typically limited to cough, bronchospasm, or transient throat irritation [8, 9]. This was further supported by a 2023 meta‐analysis on the safety of nebulized LAmB, reporting no serious adverse effects, reinforcing its potential for both prophylactic and therapeutic applications [7]. In our patient, no respiratory or systemic adverse events were observed. The favorable safety profile and targeted airway delivery could lead to other benefits such as reduced resource use and shorter hospital stay, although evidence of this is still limited. Our patient showed rapid clinical improvement following the initiation of nebulized LAmB without systemic complications, suggesting its efficacy as a therapeutic option when first‐line systemic azoles do not achieve therapeutic levels or are contraindicated or poorly tolerated. However, given the prior use of voriconazole and posaconazole, a potential cumulative antifungal effect cannot be ruled out [7].

While optimal dosing and frequency require further refinement, our case highlights the role of nebulized LAmB in patients outside the traditionally immunosuppressed spectrum of IPA. LAmB represents an effective and valuable addition to the therapeutic arsenal for tracheobronchial aspergillosis and has shown adequate prophylactic and local control, optimal safety profile and tolerability, and reduced systemic impact that can potentially improve patient‐centered outcomes. Further investigation into its role is warranted in clinical practice.

## Funding

The authors have no funding to disclose.

## Conflicts of Interest

The authors declare no conflicts of interest.

## Data Availability

The data that support the findings of this study are available on request from the corresponding author. The data are not publicly available due to privacy or ethical restrictions.
